# Abdominal obesity and low physical activity are associated with insulin resistance in overweight adolescents: a cross-sectional study

**DOI:** 10.1186/1471-2431-14-258

**Published:** 2014-10-10

**Authors:** Claudia-María Velásquez-Rodríguez, Marcela Velásquez-Villa, Leidy Gómez-Ocampo, Juliana Bermúdez-Cardona

**Affiliations:** Research Group in Food and Human Nutrition, Universidad de Antioquia (UdeA), Calle 70 No. 52-21, Medellín, Colombia

**Keywords:** Insulin resistance, Abdominal obesity, Metabolic syndrome, Physical activity, Adolescents

## Abstract

**Background:**

Previous studies have assessed the metabolic changes and lifestyles associated with overweight adolescents. However, these associations are unclear amongst overweight adolescents who have already developed insulin resistance. This study assessed the associations between insulin resistance and anthropometric, metabolic, inflammatory, food consumption, and physical activity variables amongst overweight adolescents.

**Methods:**

This cross-sectional study divided adolescents (n = 120) between 10 and 18 years old into 3 groups: an overweight group with insulin resistance (O + IR), an overweight group without insulin resistance (O-IR), and a normal-weight control group (NW). Adolescents were matched across groups based on age, sex, pubertal maturation, and socioeconomic strata. Anthropometric, biochemical, physical activity, and food consumption variables were assessed. Insulin resistance was assessed using homeostatic model assessment (HOMA Calculator Version 2.2.2 from ©Diabetes Trials Unit, University of Oxford), and overweight status was assessed using body mass index according to World Health Organization (2007) references. A chi-square test was used to compare categorical variables. ANOVAs or Kruskal-Wallis tests were used for continuous variables. Multiple linear regression models were used to calculate the probability of the occurrence of insulin resistance based on the independent variables.

**Results:**

The risk of insulin resistance amongst overweight adolescents increases significantly when they reach a waist circumference > p95 (OR = 1.9, CIs = 1.3-2.7, p = 0.013) and watch 3 or more hours/day of television (OR = 1.7, CIs = 0.98-2.8, p = 0.033). Overweight status and insulin resistance were associated with higher levels of inflammation (hsCRP ≥1 mg/L) and cardiovascular risk according to arterial indices. With each cm increase in waist circumference, the HOMA index increased by 0.082; with each metabolic equivalent (MET) unit increase in physical activity, the HOMA index decreased by 0.026.

**Conclusions:**

Sedentary behaviour and a waist circumference > p90 amongst overweight adolescents were associated with insulin resistance, lipid profile alterations, and higher inflammatory states. A screening that includes body mass index, in waist circumference, and physical activity evaluations of adolescents might enable the early detection of these alterations.

## Background

The number of adolescents who are overweight is increasing [1, 2], which is a public health problem. This status is associated with the globalisation and technological advances that affect lifestyle [3, 4]. In Colombia, the prevalence of overweight adolescents was 10.3% in 2005 [5], and this figure increased to 17.5% in 2010 [6]. The current study examines adolescents from the city of Medellin, where an overweight prevalence of 20.8% was reported in 2010 [7].

Obesity, specifically abdominal obesity, triggers insulin resistance (IR) because excessive free fatty acids and inflammatory substances alter insulin receptor signalling in different organs [8, 9]. Furthermore, IR causes the metabolic alterations that comprise metabolic syndrome (MS) [8–12]. The prevalence of MS increases with obesity [2, 13, 14].

MS has been detected in younger people at an increasing rate [10, 15, 16]. A recent study in Medellin that included 851 adolescents between 10 and 18 years old revealed rates of 25%, 4.1%, and 4.9% for overweight, MS, and IR, respectively [11]. The biological findings associated with this disease suggest that the β-pancreatic cells of these adolescents are forced to produce more insulin to maintain normoglycaemia, which predisposes them to hyperglycaemia and Diabetes Mellitus II (DM2) [12, 17, 18]. Early IR detection amongst obese adolescents might enable the application of preventive measures to decrease chronic disease development [19].

According to a recent publication, biochemical markers such as high-sensitivity C-reactive protein (hsCRP) and fasting insulin might enable early IR detection; the authors of this study suggested using hsCRP as an inflammation marker for risk stratification and treatment initiation amongst adults with moderate cardiovascular disease (CVD) risk [1]. However, this marker cannot be used for adolescents because little evidence supports the associations amongst hsCRP, the long-term risk of IR, and the development of chronic diseases during adulthood. Moreover, the authors suggested that daily hsCRP or insulin measurements are not feasible for the healthcare systems of developing countries because of the large number of adolescents [1]. Nevertheless, efforts are needed to discover anthropometric, clinical, or biochemical markers of IR for adolescents. These markers must be able to predict the progression of CVD during adulthood.

Waist circumference (WC) can be used to evaluate visceral fat, particularly amongst overweight adolescents. This measurement is correlated with obesity-related metabolic changes [20–22] and might serve as an early marker of chronic disease. However, the health institutions in Colombia do not account instruments to measure the WC and the health personnel does not register this information at the clinic history, that is why the health system just measures weight and height to classify overweight by body mass index (BMI).

Defining overweight as the consequence of a positive energy balance [23, 24], WC might not be sufficient for use as a unique predictor of chronic disease. Therefore, identifying the type of lifestyle associated with IR development amongst overweight adolescents is essential.

Many studies have characterized the metabolic and inflammatory alterations, as well as the lifestyles, of overweight adolescents [25–34]; however, not all overweight individuals are at the same risk of developing future disease. This study examined adolescents who have already developed IR because of their weight status and assessed the associations between IR and the anthropometric, metabolic, inflammatory, food consumption, and physical activity (PA) variables in overweight adolescents.

## Methods

### Study design

The study is a cross-sectional study.

### Participants

A subsample of adolescents (n = 120) between 10 and 18 years old was selected from 851 participants of the cross-sectional study “Variations in the Prevalence of Metabolic Syndrome in Adolescents According to Different Criteria Used for Diagnosis: Which Definition Should Be Chosen for This Age Group? [11]. Study conducted between 2011 and 2012.

Sample size estimations were determined in consultation with a statistician and based on data over the minimum differences expected in insulin and triglycerides (TG) values were obtained from two published studies [35, 36]. According to the analysis criteria, a three-group comparison was conducted with an alpha level of 0.05 and 80% statistical power. The sample size was calculated as 40 participants per group, and Primer® software was used for all analyses.

Three groups were formed: 1) overweight with IR (O + IR), in which BMI scores were > p85 and HOMA index values ranged from 3.2-7.1; 2) overweight without IR (O-IR), in which BMI scores were > p85 and HOMA index values ranged from 0.5-2.9; and 3) normal weight (NW) in which BMI scores ranged between p15 and p85 and HOMA index values ranged from 0.4-2.4. The groups were matched with regard to age, sex, pubertal maturation, and socioeconomic stratum.

Adolescents who used hypolipidaemic, antihypertensive, or hypoglycaemic drugs; who used corticosteroid treatments or thyroid hormones; or who consumed functional foods were excluded from this study. Adolescents with DM1, genetic diseases, or physical limitations that inhibited anthropometric measurements, as well as elite athletes and young pregnant or lactating women, were also excluded.

This study was defined as minimum risk according to the Colombia Ministry of Health, decision 008430, article 11, October 1993. The University of Antioquia Research Bioethics Committee (SIU) approved this project. Both adolescents and their parents signed an informed consent form that included the Helsinki declaration.

### Measures

#### Socioeconomic strata

Socioeconomic strata were defined based on the National Administrative Department of Statistics (DANE, in Spanish) [37] as low (strata 1 and 2), medium (strata 3 and 4), and high (strata 5 and 6).

#### Anthropometric evaluations

Weight, height, triceps fat folds (TFFs), subscapular fat folds (SFFs), and WC were measured using international tools and techniques [38, 39]. Nutritional status was classified as NW (BMI between p15 and p85) and overweight (BMI > p85) based on WHO (2007) [40, 41]. WC scores > p90 were considered high according to the third National Health and Nutrition Examination Survey in the United States (NHANES III) [38]. Total body fat percentage (%BF) was calculated using TFFs and SFFs according to the Lohman equation: **Σ folds >35 mm** %BF = 0.783 ΣTFFs, SFFs + 1 (men) and %BF = 0.546 ΣTFFs, SFFs + 9.7 (women); **Σ folds <35 mm** %BF = 1.21(ΣTFFs, SFFs)-0.008 (ΣTFFs, SFFs)^2^ + 1(men) and %BF = 1.33 (ΣTFFs, SFFs)-0.013 (ΣTFFs, SFFs)^2^ + 2.5 (black women: 2.0 and white women: 3.0). Obesity was classified as %BF >25% for boys and >32% for girls, NW was classified as 12-25% for boys and 15-32% for girls [39].

#### Arterial pressure measurements

Blood pressure was measured using a mercury sphygmomanometer (Riester®) and bracelets appropriate for adolescents. Blood pressure measurements ≥ p90 based on age, sex, and height were considered high according to the fourth task force [42].

#### Pubertal maturation

Pubertal maturation was evaluated via self-report and classified according to Tanner [43, 44].

#### Biochemical tests

Blood from the antecubital vein was drawn after a 10- to 12-hr period of fasting. Serum was isolated and stored at -80°C.

##### Serum Lipoproteins

TC, HDL-c, LDL-c, and TG were measured by spectrophotometry in a RA50 (Bayer, series 71663) photocolourimeter using specific kits (BioSystems Reagents and Instruments). The cut-off points for the diagnosis of lipid profile alterations were TC ≥200 mg/dL, LDL-c ≥130 mg/dL, HDL-c <40 mg/dL, and TG ≥130 mg/dL [45]. Arterial indices were calculated using the ratios between lipid fractions. According to the arterial index (AI: LDL-c/HDL-c), scores >3.5 and >3 were considered high risk for men and women, respectively; according to the Castelli index (CI: CT/HDL-c), scores >5 and >4.5 were considered high risk for men and women, respectively [46].

##### Glycaemia and Insulinaemia

Standardised colourimetric enzymatic methods were used. Plasma insulin was measured using an automated microparticle enzyme immunoassay (MEIA). IR was estimated using homeostatic model assessment (HOMA) via the HOMA Calculator ©, Version 2.2.2 (Diabetes Trials Unit of University of Oxford). Reference values for adolescents do not exist for HOMA-estimated IR; however, IR was defined as a value >3.1 based on three findings: 1) the p95-HOMA value was 3.1 in the reference study of 851 adolescents [11]; 2) the cut-off published by Lee JM et al. in 2006 [47]; and 3) Yin et al. [48] confirmed the cut-off to be 3.1 for IR classification amongst children and adolescents [48]. Hyperglycaemia was defined amongst adolescents as fasting glucose >110 mg/dL [26].

##### High-sensitivity CRP (hsCRP)

hsCRP was determined using immunoturbidimetry. Cardiovascular risk was considered low when the score was <1 mg/L, medium when the score was between 1 and 3 mg/L, and high when the score was >3 mg/L [49].

#### Food consumption

To obey with the goal to evaluate calories and nutrients intake for one person by day, a 24-hours recall was randomly distributed during different weekdays. A second questionnaire was also distributed amongst a random subsample constituted by 20% of the study population (24 adolescents) to calculate intra-individual variation [50, 51]. The dietary intake evaluation program (EVINDI v4) was used [52–55]. Nutrient reports were generated using the PC version of the Software for Intake Distribution Estimation (SIDE) program, Iowa State University, version 1.0, June 2004.

#### Physical activity

The 3-day physical activity recall (3DPAR) method was applied [56]. MET values for each activity were calculated based on the American College of Sports Medicine Compendium of Physical Activities [57]. Physical activity of 3–6 METs was classified as moderate-to-vigorous (MVPA), and >6 METs was classified as vigorous (VPA) [58]. Adolescents were categorised into three PA levels: sedentary (no MVPA or VPA), active (1 or more MVPA per day), and very active (1 or more VPA per day) [57].

#### Time spent watching television/playing video games

Time reported was converted into hours per day and categorised into 2 groups: less than 3 hours and 3 or more hours per day [5].

### Statistical analyses

The Shapiro-Wilk test was used to test the normality of continuous variables; ANOVAs (with post hoc Scheffé test) and Kruskal-Wallis tests were used to assess the differences amongst the 3 groups, and Student’s t-test and the Mann–Whitney U test were used for 2-group comparisons. Chi-square tests were used to calculate the association between categorical variables. Pearson’s and Spearman’s coefficient were used to assess the correlations between variables. The probability of IR occurrence was calculated by Odds ratios (ORs). Through multiple linear regression model was estimated the average value of HOMA (dependent variables) according to the presence of independent variables (hsCRP, WC and PA). Scatterplots, ANOVAs, R^2^ and partial beta p-values were used to evaluate goodness of fit, and model assumptions were verified. p < 0.05 was considered significant. All statistical analyses were performed using SPSS® v21.0.

## Results

No differences were observed with regard to the variables used to match groups; the mean age of adolescents was 14.2 years; 52.5% were male; 70% were post pubertal; 50% were of a medium socioeconomic stratum; and 87.5% were in high school (Table 1).

**Table 1:** **Adolescent sociodemographic characteristics by study group**

Variables	O + IR ***n = 40***	O-IR ***n = 40***	NW ***n = 40***	p
**Age in years (X ± SD)**	14.2 ± 2.2	14.2 ± 2.2	14.2 ± 2.3	0.997*
**Age group (%)**				
10-13.9	52.5	52.5	52.5	0.999**
14-18 years	47.5	47.5	47.5	
**Sex (%)**				
Men	52.5	52.5	52.5	0.999**
Women	47.5	47.5	47.5	
**Socioeconomic strata (%)**				
Low	40	37.5	37.5	0.999**
Medium	47.5	50	50	
High	12.5	12.5	12.5	
**Education level (%)**				
Primary	12.5	12.5	12.5	0.999**
High school-university	87.5	87.5	87.5	
**Pubertal maturation (%)**				
Pre-puberty	10	10	10	0.999**
Puberty	22.5	20	20	
Post- puberty	67.5	70	70	

*X ± SD ANOVA, **Chi-square.

The anthropometric variables BMI, WC, SFFs, and %BF (Table 2) were significantly higher in the O + IR group than in the O-IR group; furthermore, the O-IR group had significantly higher values than the NW group. Approximately 82.5% of the adolescents in the O + IR group were obese based on %BF.

**Table 2:** **Adolescent anthropometric characteristics by study group**

Variables	O + IR n = 40	O-IR n = 40	NW n = 40	p
WC cm (X ± SD)	86.77 ± 10.77	77.21 ± 7.49	65.29 ± 5.43	0.000^abc^*
BMI Kg/m^2^ (Me-RQ)	26.86-18.32	24.95-11.94	19.7-8.86	0.002^abc^**
%BF (Me-RQ)	34.23-40.73	31.75-42.26	24.47-21.84	0.040^abc^**
TFFs mm (X ± SD)	23.10-6.00	20.58-6.81	13.85-4.45	0.000^ac^*
SFFs mm (Me-RQ)	25.00-42	17.50-40	10.5013	0.002^abc^**

^a^Differences between NW and O-IR, ^b^differences between O + IR and O-IR, ^c^differences between NW and O + IR.

*ANOVA.

**Kruskal-Wallis.

WC: waist circumference.

BMI: body mass index.

%BF: body fat percentage.

TFFs: triceps fat folds.

SFFs: subscapular fat folds.

All adolescents in the O + IR group had a WC >75p. Significantly more participants (25%) had high WCs (>90p) in the O + IR group compared with the O-IR and NW groups (p = 0.00001). Overweight adolescents who also presented high WCs were 1.9 times more likely to develop IR (OR = 1.9, CIs = 1.3-2.7, p = 0.013). WC was correlated with HOMA (r = 0.67, p = 0.00001).

The O + IR group had higher HOMA values than the other groups (M_e_ = 3.85; p = 0.0001); however, no differences or IR indicative values were observed between the O-IR and NW groups (HOMA: M_e_ = 1.3 and M_e_ = 0.95, respectively; Table 3).

**Table 3:** **Adolescent clinical and biochemical variables by study group**

Variables	O + IR n = 40	O-IR n = 40	NW n = 40	p*
HOMA (Me-RQ)	3.85-0.85	1.30-1.08	0.95-0.70	0.0001^ab^
TC (Me-RQ)	192.5-211	175-189	165-178	0.942
TG (Me-RQ)	149-401	101-328	81.5-308	0.015^ab^
HDL-c (Me-RQ)	39-42	49-61	50-60	0.001^ab^
LDL-c (Me-RQ)	100-142	92-145	87.5-95	0.470
AI (Me-RQ)	2.67-1.18	2.06-0.83	1.76-0.62	0.015^ab^
CI (Me-RQ)	4.44-5.7	3.69-4.0	3.53-5.2	0.005^ab^
hsCRP (Me-RQ)	1.32-15.94	0.81-12.65	0.39-15.78	0.020^ab^
DBP (Me-RQ)	67-39	67-33	62-31	0.954
SBP (Me-RQ)	111-56	110-39	100.5-33	0.342

^a^differences between O + IR and O-IR, ^b^differences between NW and O + IR.

*Kruskall-Wallis.

TC: total cholesterol.

TG: triglycerides.

HDL-c: high-density lipoprotein.

LDL-c: low-density lipoprotein.

AI: arterial index (LDL-c/HDL-c).

CI: Castelli index (TC/HDL-c).

hsCRP: high-sensitivity C-reactive protein.

DBP: diastolic blood pressure.

SBP: systolic blood pressure.

Inflammatory status, as assessed by hsCRP (Table 3), was significantly higher in the O + IR group than in the other groups (p = 0.020); this group also showed the highest percentage of adolescents with high cardiovascular risk (22.5%; hsCRP >3 mg/L). Furthermore, these participants were 1.6 times more likely to have inflammation (hsCRP ≥1 mg/L) than the O-IR group (OR = 1.6, CIs = 1.0-2.7, p = 0.028). hsCRP was significantly correlated with HOMA (r = 0.35, p = 0.0001).

TG and HDL-c significantly differed between the O + IR and other groups (p = 0.015 and p = 0.001, respectively). Approximately 60% of the adolescents in the O + IR group simultaneously presented low HDL-c and high TG values. HOMA was negatively correlated with HDL-c but positively correlated with TG (r = -0.31, p = 0.0010 and r = 0.45, p = 0.0001, respectively). The O + IR group was 2.1 times more likely to present low HDL-c (OR = 2.1, CIs = 1.4-3.3, p = 0.001) and 1.7 times more likely to present high TG (OR = 1.7, CIs = 1.1-2.7, p = 0.012) than the O-IR group.

The AI and CI were significantly higher for the O + IR group than for the other groups (p = 0.015 and p = 0.005, respectively; Table 3); both indices were significantly correlated with HOMA (AI: r = 0.36 p = 0.00001, CI: r = 0.32 p = 0.0001). The probability of presenting high AI and CI values was increased by 2 (OR = 2, CI = 1.4-2.8, p = 0.006) and 1.6 (OR = 1.6, CIs = 1.1-2.4, p = 0.026) for the O + IR group compared with the O-IR group.

The average caloric consumption was 2197 kcal/day, with a macronutrient distribution of 13% protein, 55% carbohydrates (CHO), and 32% fat. Of the total fat, 13% was saturated, 11% was monounsaturated, and 7% was polyunsaturated, with no differences between groups. Both overweight groups consumed more fast food; the O + IR group consumed the fewest fruits and vegetables.

Adolescents in the O + IR group performed significantly less PA (measured as METs/day) than those in the O-IR group (p = 0.043); furthermore, PA was negatively correlated with HOMA (r = -0.22, p = 0.016). Approximately 46% of the O + IR group was sedentary, and 72.5% watched more than 3 hours of TV per day. These differences were statistically significant when compared with the other groups (p = 0.033). Approximately 45% of the O + IR group had low PA values, compared with the 47.5% of the NW group who were active (p = 0.018; Table 4). Within the overweight groups, participants who watched TV for 3 or more hours per day were 1.7 times more likely to develop IR (OR = 1.7, CIs = 0.98-2.8, p = 0.033) than those who watched less TV.

**Table 4:** **METs and PA classification within the study groups**

Variables physical activity	O + IR n = 40	O-IR n = 40	NW n = 40	p
**METs/day (Median-RQ)**	58.83 ± 46.2	63.75 ± 52.66	63.48 ± 35.33	0.043^a^*
**Average hours TV/day (Median-RQ)**	4 ± 10	2.75 ± 8.1	3 ± 9.01	0.035^a^*
**PA Classification by PA groups (%)**				
Sedentary	46	15	27.5	0.018^a^**
Low Active	28	45	25	
Active (%)	26	40	47.5	
**Watch TV ≥ 3 hours (%)**	72.5	50	52.5	0.033^ab^**

^a^differences between O + IR and O-IR, ^b^differences between NW and O + IR.

*Kruskal-Wallis, **Chi-square.

In the initial exploratory data analysis, the variables that show association with HOMA were tested in a multiple linear regression model, in the process were introduce the variables one by one and the variable that was not significant was discarded. Multiple linear regression model explained 43.3% of the variance in the associations between HOMA and hsCRP, WC, and PA. However, only WC and PA significantly explained HOMA. For every 1 cm increase in WC, the HOMA index increased by 0.082, and for every MET increase in PA, the HOMA index decreased by 0.026. This model fulfilled the assumptions of linearity, normality, constant variance, independence and collinearity (Table 5).

**Table 5:** **Multiple linear regression between HOMA index and IR-explicatory variables**

Variables	β	CIs	p	VIF
hsCRP (mg/L)	0.01	-0.06-0.08	0.707	1.007
METs/day	-0.03	-0.05 - -0.01	0.013	1.004
WC (cm)	0.08	0.06-0.10	0.00001	1.011

R^2^: 0.433; normality: p = 0.332.

ANOVA: 0.00001; OR: 45% (0.448).

Dependent variable: HOMA.

VIF: Variance Inflation Factor.

## Discussion

The current study showed that overweight adolescents with IR differ from those without IR with regard to their bodily dimensions and lifestyles. Overweight adolescents with WCs > p90 and less PA (METs/day) are more likely to have IR.

The O + IR group had higher BMI, WC, and %BF values than the O-IR and NW groups (Table 2). These adolescents also presented higher HOMA index values (Me = 3.85), higher inflammatory statuses (hsCRP = 1.32), and more metabolic alterations (lower HDL-c, higher TG) and higher indices of arterial risk than those in the O-IR and NW groups. The above findings were statistically significant. Such findings confirmed that MS alterations are triggered amongst overweight adolescents who also present IR.

Not all overweight individuals are at the same risk of developing future disease. This subgroup has been called the “healthy obese” [1]. The current study did not find significant differences with regard to metabolic alterations, lipid profile (HDL-c and TG), glycaemia, insulin, the HOMA index, arterial indices, or inflammatory variables (hsCRP) between the O-IR and NW groups. These results suggest that the disease risk for these adolescents is not directly associated with BMI. The connection between overweight status and metabolic alterations seems to be in the development of IR due to excess visceral fat.

Within the overweight groups, IR development was directly associated with upper segment fat, based on WC and SFFs values. These values were significantly higher in the O + IR group than in the O-IR group; moreover, according to the multiple regression model, WC was the only marker that (together with sedentarism) explained 43.3% of the occurrence of IR amongst overweight adolescents. Kotlyarevska K et al. reported similar results with regard to 12- to 18-year-old adolescents. These results showed that BMI and WC were associated with IR as measured by the HOMA index [22]; the same results were previously reported for adults [20]. This association can be explained by the proinflammatory adipokines and the release of non-esterified (free) fatty acids from visceral fat to the portal system. These compounds induce lipotoxicity and insufficient phosphorylation (serine phosphorylation) in the insulin type 1 receptor (IRS-1) substrate present in adipocytes and myocytes [8, 59]. This effect results in insulin signalling alteration and IR, which in advanced states, leads to DM2 due to β-cells failure [12].

Proinflammatory adipokine production also triggers a mild and chronic inflammatory state that leads to CVD [8]. In the current study, the O + IR group was 1.6 times more likely to develop CVD risk than the O-IR group, as measured by hsCRP. In accordance with these results, Saíto E et al. found that an increase in the WCs of 10- to 13-year-olds was significantly associated with IR in both genders and with an increase in hsCRP amongst male adolescents [60]. Adiposity and IR increased the activation and aggregation of thrombocytes, promoted smooth muscle cell proliferation, increased adhesion molecule expression, and decreased nitric oxide bioavailability in the endothelium. All of these effects produce pro-atherosclerotic alterations in the arterial wall that affect cardiovascular health [61].

The O + IR group showed higher plasma TG and lower HDL-c concentrations than the other groups. Koike T et al. found similar results amongst young obese adults with IR [20]. These lipid alterations are related to an excess release of fatty acids from adipocytes and hepatic IR. Both situations increase TG synthesis [62]. At the same time, hepatic lipase (HL) and cholesterol ester transfer protein (CETP) increase, which increase HDL_2_-c hydrolysis and the formation of much smaller HDL_3_-c particles. The latter are excreted by the kidney, thereby decreasing their concentration [63]. Lipid profile, arterial, and CI alterations, as well as high hsCRP concentrations, in the O + IR group indicated a higher future risk of CVD amongst young people [64].

Findings concerning the lifestyle characteristics that affect IR occurrence and its complications were of great importance with regard to the objectives of the present study [3, 12]. Diet composition is undoubtedly related to health risk factors [26]. Our study showed that the groups had similar caloric intakes (2197 kcal/day); however, these intakes did not have the same effect on each group because of the differences in PA levels. This finding might explain the nutritional status differences observed amongst groups because the same caloric intake might be recommended for active adolescents (NW) but not for sedentary adolescents (O + IR) for whom the same caloric intake might represent an energy surplus.

Energy imbalances that lead to overweight individuals are directly correlated with insulin metabolism alterations [65]. Androutsos O et al. and Mirza N et al. evaluated lifestyle and IR associations in children younger than 15 years old in Greece and the United States. They showed that a higher consumption of sweetened beverages and more time watching television were positively associated with IR [25, 28]. The present study also found that adolescents with O + IR had significantly less PA (measured as METs) and they watched more television than the other groups (p = 0.043 and p = 0.035, respectively). In addition, an inverse relationship was found between the HOMA index and METs/day; furthermore, the overweight adolescents who watched TV ≥3 hours/day were 1.7 times more likely to develop IR.

Although adolescents with O-IR had significantly higher BMIs,%BF, and WCs than NW adolescents, they had similar PA (METs/day) levels and watched a similar number of hours of TV. However, they had more PA and watched less TV than O + IR adolescents. These findings suggest that PA protects adolescents from IR development, regardless of their overweight status. In accordance with this result, the regression analysis showed that for each MET, the HOMA index decreased by 0.026.

A recent meta-analysis [66] showed that exercise/training had a small-to-moderate effect on fasting insulin and improved IR in youth (Hedges effect size = 0.48 [95% CIs: 0.22–0.74], p = 0.001, and 0.31 [95% CIs: 0.06–0.56], p = 0.05, respectively). Our group performed another study showing that a PA intervention amongst 10- to 18-year-old adolescents, together with the daily consumption of 5 portions of fruits and vegetables, significant decreased WC, glycaemia, insulinaemia, and HOMA-IR [29]. In summary, these and other studies concluded that lifestyle changes including PA (regardless of its intensity, duration, and type) hamper IR progress in youth, primarily amongst those who are overweight and have adiposity, regardless of pubertal maturation and gender [67–74].

Healthy lifestyle habits should be favoured to prevent and treat these complications. PA models should be included in schools that meet the recommendations of 1 hour/day of MVPA to maintain active adolescents who are in good health [75]. At an individual level, adequate dietary habits and active lifestyles [65] should be adopted. If these habits are maintained from childhood through adulthood, then they might decrease organs damage and DM2 as well as CVD morbidity and mortality rates [75].

This study has certain methodological limitations. For example, although the 24-hr food consumption questionnaire properly reflects population food consumption, it does not enable the establishment of individual associations amongst biochemical variables due to the high intra-individual variability of this variable. On the other hand, PA was assessed in young people using the method 3DPAR, because the study did not have a better instrument like accelerometer. However, Cheryl B [74], in their validation study of the method PDPAR, demonstrated moderate correlations between this questionnaire and the accelerometer MTI, similar or higher to the correlations found with another self-report measures.

## Conclusions

The results of the present study suggest that a WC > p90 and sedentary behaviour are associated with IR, lipid profile alterations, higher inflammatory status, and CVD risk amongst overweight adolescents. This two variables associated with HOMA, could be considered in the assessment of adolescents with overweight during the process of attention in health to detect those that required confirmatory laboratory tests of IR and to perform multidisciplinary interventions that modify the risk factors in adolescents with overweight, based on the eating habits and healthy lifestyles.

Future studies should also consider looking at a broad age range – from preschool through young adulthood - to determine how a proper screening including BMI, WC and PA evaluations might enable the early detection of features that promotes the development of chronic diseases like diabetes and CVD. This evaluation could have effects not only on the individual but on the health economy with the completion of paraclinical only young people who are at risk.

## Authors’ information

^1^ ND, Mg Basic Biomedical Sciences. Professor, Universidad de Antioquia, Human Nutrition and Food Research Group Leader, Universidad de Antioquia, Medellin, Colombia

^2^ ND Human Nutrition and Food Research Group, Universidad de Antioquia, Medellin, Colombia

^3^ ND Human Nutrition and Food Research Group, Universidad de Antioquia, Medellin, Colombia

^4^ ND, Mg Food Science and Human Nutrition. Human Nutrition and Food Research Group, Universidad de Antioquia, Medellin, Colombia

## Abbreviations

IR: Insulin resistance
MS: Metabolic syndrome
DM2: Diabetes mellitus 2
hsCRP: High-sensitivity C-reactive protein
CVD: Cardiovascular disease
WC: Waist circumference
PA: Physical activity
BMI: Body mass index
SFFs: Subscapular fat folds
TFFs: Triceps fat folds
TC: Total cholesterol
HDL-c: HDL cholesterol
LDL-c: LDL cholesterol
TG: Triglycerides
AI: Arterial index
CI: Castelli index
MVPA: Moderate-to-vigorous physical activity
VPA: Vigorous physical activity
MET: Metabolic equivalents
%BF: Body fat percentage
OR: Odds ratio
CIs: Confidence intervals.
